# Information Management in Healthcare and Environment: Towards an Automatic System for Fake News Detection

**DOI:** 10.3390/ijerph17031066

**Published:** 2020-02-08

**Authors:** Pablo Lara-Navarra, Hervé Falciani, Enrique A. Sánchez-Pérez, Antonia Ferrer-Sapena

**Affiliations:** 1 Ciencias de la Información y de la Comunicación, Universitat Oberta de Catalunya, 08035 Barcelona, Spain; plara@uoc.edu; 2 Tactical Whistleblower Association, 46022 València, Spain; herfal@upvnet.upv.es; 3 Instituto Universitario de Matemática Pura y Aplicada, Universitat Politècnica de València, 46022 València, Spain; easancpe@mat.upv.es

**Keywords:** healthcare, environment, fake news, reinforcement learning, graph

## Abstract

Comments and information appearing on the internet and on different social media sway opinion concerning potential remedies for diagnosing and curing diseases. In many cases, this has an impact on citizens’ health and affects medical professionals, who find themselves having to defend their diagnoses as well as the treatments they propose against ill-informed patients. The propagation of these opinions follows the same pattern as the dissemination of fake news about other important topics, such as the environment, via social media networks, which we use as a testing ground for checking our procedure. In this article, we present an algorithm to analyse the behaviour of users of Twitter, the most important social network with respect to this issue, as well as a dynamic knowledge graph construction method based on information gathered from Twitter and other open data sources such as web pages. To show our methodology, we present a concrete example of how the associated graph structure of the tweets related to World Environment Day 2019 is used to develop a heuristic analysis of the validity of the information. The proposed analytical scheme is based on the interaction between the computer tool—a database implemented with Neo4j—and the analyst, who must ask the right questions to the tool, allowing to follow the line of any doubtful data. We also show how this method can be used. We also present some methodological guidelines on how our system could allow, in the future, an automation of the procedures for the construction of an autonomous algorithm for the detection of false news on the internet related to health.

## 1. Introduction: Social Networks in the Context of Health

The analysis of social media—in particular, the measurement of their influence on users—is attracting greater importance from several points of view, including academic and economic perspectives. To an increasing degree, social media networks determine the opinions of a large portion of the population, hence the interest in carrying out studies, especially in marketing, on how people are drawn to the choice of a brand, a commercial product or a political leader. In fact, it has recently become widely known that a great deal of information is manipulated to influence public opinion on political issues in processes of global significance.

In health, social media are also opinion formers. In fact, content created on the web has the potential to provide good information as well as generate opinions and social behaviours that in some cases may become dangerous. For example, the anti-vaccination movement, which continues to have a significant impact in the area of public health, has become a relevant problem in some countries. This wave of social action has generated the return in Brazil of some diseases that had been eradicated, or of diseases that had become rare, such as measles (see Reference [1] and the references therein). This state of public opinion has generated what is called vaccine hesitancy—that is, delay in acceptance or rejection despite having the recommended vaccines available in health services—which is a serious public health problem that is promoted through social networks.

Issues related to alternative treatments for cancer are other examples. The “preachers” of such treatments recommend remedies including the ingestion of bleach or herbs that may not be beneficial to health (see, for example, References [2,3]). Alternative treatments for medical problems based on homeopathy—considered a pseudoscience by many—have also spread, even though the products they recommend have not been adequately tested. All these new issues are finding a very easy way of dissemination in internet, by means of the use of histagram or twitter.

In recent years, healthcare institutions have launched a number of initiatives to integrate social media platforms in an attempt to generate a greater relationship between the information needs of patients and the knowledge of medical doctors and researchers. The experience of an Italian health organization provides one example [4]. For this project, the contents of the comments, information and videos published on the Facebook pages of this organization were analysed. Despite expectations that the use of social media would provide more patient-centred care, there is little evidence of health benefits produced by patient use of social media or platforms through which health professionals and patients share knowledge and experience [5]. Various studies also indicate that public health professionals must identify the information needs of patients and provide them with an appropriate service in this regard [6]. Influencers in this type of network are vital for the network to be effective and sufficiently known. Users would probably use them more than other unreliable networks because of the qualified information they provide. However, unqualified networks tend to be more effective, since they have good community managers and are often successful in conveying information.

**Figure 1:**
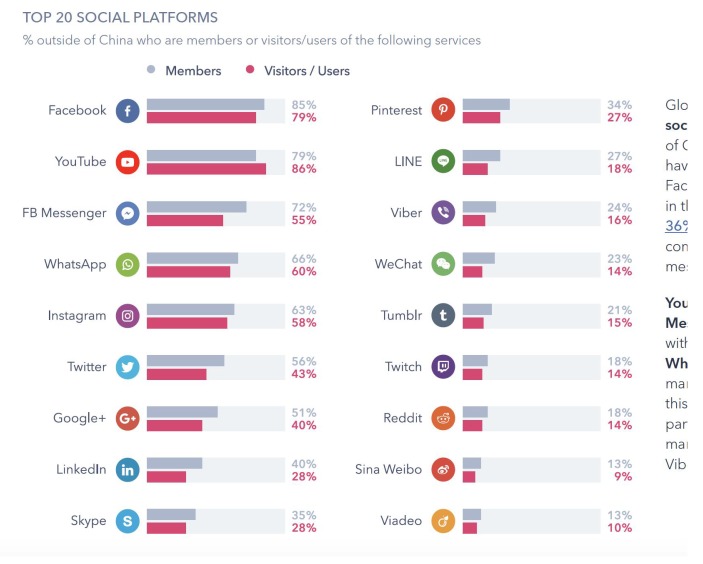
Users of social media. Source: Report 2018. Global Web Index. Report in social media.

**Figure 2:**
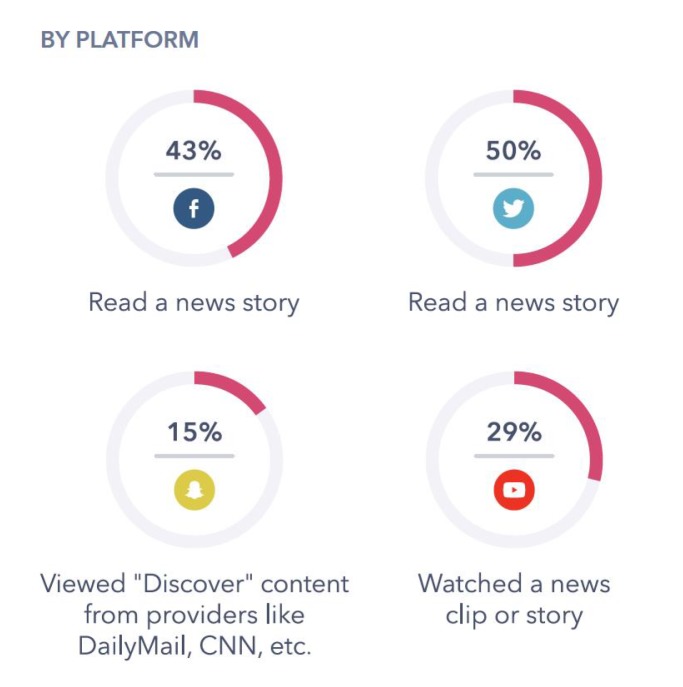
Reads. Source: Report 2018. Global Web Index. Report in social media.

Since the American election campaign won by Donald Trump, the dissemination of false information through the web has grown exponentially (see for example Reference [7]). The reader can find a complete review on works that evidence this fact in Reference [8]. A similarly large increase has also been observed in information related to pseudoscience. Facebook in particular is the medium in which this misleading information—fake news, alternative news or pseudoscience-related information—has proliferated the most [9]. It should be borne in mind that this social network is not a natural channel for the dissemination of scientific results, and scientists rarely use it to publicize the specialized content of their research. Perhaps the use of this network by scientific societies, public organizations or other institutions could contribute to increasing the quality of the information. According to the market research company GlobalWebIndex, use of major social media platforms during 2018 was as shown in Figure 1 (see also Figure 2). Another observation is that it is the youngest members of the population—aged between 16 and 24 years old—who spend the most time using these networks (more than three hours a day). This group is also the one that uses Instagram the most [10].

This article aims to provide a methodological approach to the problem of “cleaning up” specialized health- and environment-related information. It proposes a heuristic tool to analyse the behaviour of users of Twitter, the social network with the greatest impact in this respect. Our goal is to provide a method for graphically showing how information is naturally disseminated via this network and how an analyst use our system to detect fake news and remedies for diseases that are unsupported by science. Thus, we present a method for the construction of a dynamic database based on graphs of general information obtained from the internet (Twitter, other social media and web pages). Supported by the graph database management system Neo4j (see Reference [11]), which is well-known for its use in analysing financial data, our method allows us to preserve the direction of information flow and general relational information based on the natural way in which tweets or other internet information item types are diffused. Due to the technical properties of our procedure, we believe that it can also provide the basic structure for the future development of a stand-alone system for the automatic detection of false news and inaccurate content using an unsupervised algorithm.

After this introduction, Section 2 is devoted to explain the methodology of our study, which is mainly related to the construction of a database of tweets and the explanation of how to use it. It contains three subsections, in which we present the objective of the study (Section 2.1), the methodological purpose (Section 2.2) and the methods (Section 2.3). Section 3 is dedicated to presenting our results, and Section 4 its discussion. Finally, in Section 5 we present the conclusions, also explaining how our method could constitute a new step for the automatic detection of inaccurate or false information related to health.

As a final observation, we must say that this study is mainly methodological and does not intend to provide specific information on any particular public health issue. Furthermore, although the final objective of our research program is to provide an algorithm for the detection of fake news in the field of public health, we present here a first step that does not allow such an automatic use, but the structured database that could be at the base of such a tool. At this stage, the program provides a framework for the visualization of chains of tweets, and therefore a heuristic tool for analysts to detect false information.

## 2. Methodology

### 2.1. Aim of the Study

As we have explained, information appearing on the internet and on social media sways opinion concerning potential remedies for diagnosing and curing diseases. This has repercussions on the health of citizens and affects medical professionals who, in many cases, have to justify both the diagnoses they make and the treatments they propose. In abstract terms, it can be said that the dynamics of dissemination and propagation of these opinions are closely related to those of fake news within these information networks.

Our purpose is to describe a procedure for the construction of a graph database of tweets implemented in the program Neo4j following some concise rules. The final result is a database that allows an analysis of the tweets following the relational items that the analyst has decided for the construction, preserving the temporal order, the origin of the tweet, the final recipient and the interaction with other users. The program allows a friendly graphic presentation of the results, and allows navigation by simply clicking on the node (vertex) or the relationship (arrow). The new tool thus becomes a highly specialized instrument for the heuristic analysis of tweet chains. Based on the relational database thus designed, algorithms for automating searches can be easily performed. We have already experimented with some of them, although their technical description is beyond the scope of this paper. To explain the procedure we use a specific test/example of tweet chain related to the World Environment Day 2019, which is not directly related to public health issues, but the same dissemination patterns are expected.

### 2.2. Methodological Purpose

In terms of the general framework in which our specific tool is rooted, it must be said that using an unsupervised algorithm for the automatic detection of fake news is a difficult task. Although there are currently several computer-based methods that can be used for this purpose [12,13]), no well-established working procedure for the detection of inaccurate internet information exists as of yet. To some extent, the area remains an open playing field, beginning with the scope of the definition of fake news. Any analysis of current news must rely on a formal classification of what constitutes a piece of misinformation (fake news, not entirely accurate information, rumour, decontextualized information, etc.), though no such classification has yet been agreed upon. Differences between these notions of fake news are confusing, make classification difficult and lead to certain information items being incorrectly flagged as wrong. For this reason, it is necessary to specify the meaning and type of information in order to classify an item as “wrong”. Notwithstanding, our interest is not in finding a general classification procedure, but rather in constructing an automatic algorithm for identifying news items that could be false, unclear, or simply confusing for the reader. From a mathematical point of view, several concepts may be used to devise an initial semantic approach that defines rational families of related words so that a topic may be identified by determining the semantic fields of a given concept. This enables detection of information with some degree of inaccuracy.

Another possible approach consists of analysing time dynamics in the diffusion of news, using fast information transmission systems such as Twitter. For instance, it may be argued that fake news spreads faster than real news. This leads us to two methods of detecting inaccurate information via internet-based information diffusion systems.

The first involves the identification of generic properties shared by erroneous information items. These properties can be understood as intrinsic characteristics of wrong items (for example, dubious sources) or diffusion dynamics (abnormally fast diffusion, for example). The second is the result of an experimental procedure: a deep learning method based on neuronal network training can be designed using graph patterns collected from a large set of fake news. However, this does not fall within the scope of this paper; our focus is on a primary organization system for current news based on graph knowledge. In the context of so-called open-source intelligence (OSINT), we are proposing a new model for organizing data retrieved from open access free information sources, which allows us to iteratively construct a knowledge graph. This would allow in future developments—it is in fact a first necessary step, in our opinion—to build new automatic tools for fake news detection.

### 2.3. Methods

Our methodological approach builds on previous work and on a wide range of computer tools related to the use of graph theory for the design of fraud detection algorithms of a mainly financial nature (see, for example, References [12,14,15]). As we have said, the main objective of our method is to define an adequate framework to automatically detect fake news. Many mathematical and computational techniques have been broadly applied in the similar context of the exchange of accounting and financial information (an introduction to this context can be found in References [13,16,17]. For our purposes, we have developed an algorithm that automatically constructs an evolutionary graph with information on a particular topic gathered from internet sources, using the timeline as the order criterion. We have already explained why we have focused on Twitter (primary) and web pages (secondary) as the starting points for our knowledge graph, though other sources could also be used (Instagram, WhatsApp, etc.). The scientific literature already shows that, in order to obtain more in-depth results in specific frameworks, some advanced improvements could be made to the internet information collecting techniques (see Reference [18], for example, and the references therein). However, we have decided to start with a direct method that consists of collecting all the tweets related to a given topic and organizing them into a graph using Neo4j.

Broadly speaking, the vertices of the graph are the users, while the edges and arcs are produced by the relation established between users when a user posts a tweet related to a certain topic, for example. Once the primary set is defined (gathering all tweets in Spanish on a current event), it is then selectively enriched according to a certain pattern. The original set allows a secondary set of users to be defined, chosen according to a fixed rule: for example, the set of followers of the users in the primary set. This new set is used to enrich the graph, preserving the previous vertices and edges and adding new ones together with the new arcs and edges defined by them. It is worth mentioning that the Neo4j system allows specific database-built information for each vertex, arc or edge to be included in the graph, which can then be stored along with its properties.

Technically, Neo4j uses a classification method based on nodes, relations and labels. The information can be written, for example, to a csv file and then uploaded to the program with the Import function (Desktop). A database is then generated in Neo4j. The user can mark in the csv file which columns are nodes (vertices), relations, and set the labels between them. This allows to make more effective the searches, and find in our case for example the tweets that are related to a particular hashtag. Each node (for example, defined by “Authors” of tweets), relations (who sends the tweet to whom), and the tags mark how the nodes and relations are connected. Below is an example of the code we use to implement the data in the program.

load csv with headers from ’file:///data.csv’ as TWITTER
merge (authortw:Authortw{authortw:TWITTER.authortw}) (Create a node)
merge (authorrtw:Authorrtw {authorrtw:TWITTER.authorrtw}) (Create a node)
merge (authorrtw)-[:retweetto{retweetto:TWITTER.authorrtw}]->(authortw) (Create a relation)

## 3. Results. A Case Study: Fake News in the World Environment Day 2019

In this section we present a case study of our analytical method, focused on a specific set of tweets, which will allow us to establish some general rules for the systematic analysis of the dissemination of false information on Twitter. It should be noted that the scheme presented here was the result of the application of a trial and error procedure; many different changes and corrections were needed to obtain this final set of rules. Thus, in the last iteration of our investigation, we commenced the test of our structuring method using tweets in Spanish posted on World Environment Day, 5 June 2019. The procedure is described below:

- First, we detected the total set of tweets with the phrase “Día Mundial del Medio Ambiente” (World Environment Day). A knowledge graph was constructed with the original set of tweets and any interaction with them. The arrows in the graph represent the propagation direction of the tweets.
- This original set was then enriched with the tweets of the set of followers of the users involved in the primary set.
- Users followed by the people involved in the original set of tweets were also included.
- Several selective proofs were made by gathering tweets containing specific hashtags.
- The final result is an extended graph of users connected by arrows representing different properties related to World Environment Day.
- This framework enables different analyses to be performed. Usually, the analyst’s interest can be focused on following a certain hashtag, which represents the main topic he or she is interested in following.

Let us illustrate this fact with a particular case, for which we developed a complete analysis. For example, we can use it to search for interaction in the set of users that follow the hashtag “reciclaje” (recycling). We present below a possible specific analysis performed using the graph thus generated.

**Figure 3:**
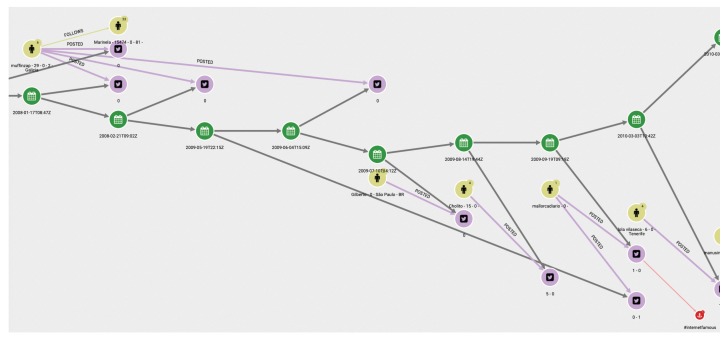
An example of a graph for the analysis of tweets: tweets and users in a timeline.

**Figure 4:**
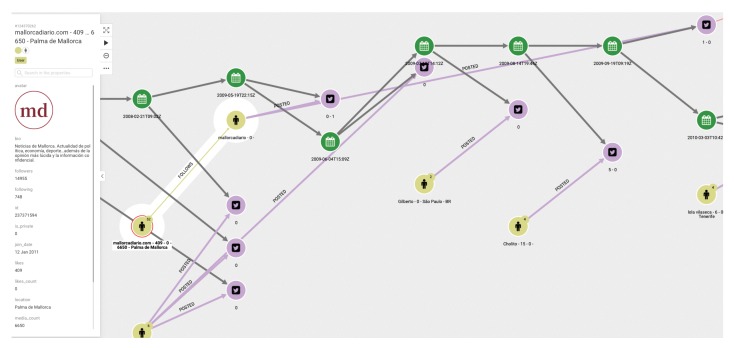
Analysis of tweets: relationship among users.

**Figure 5:**
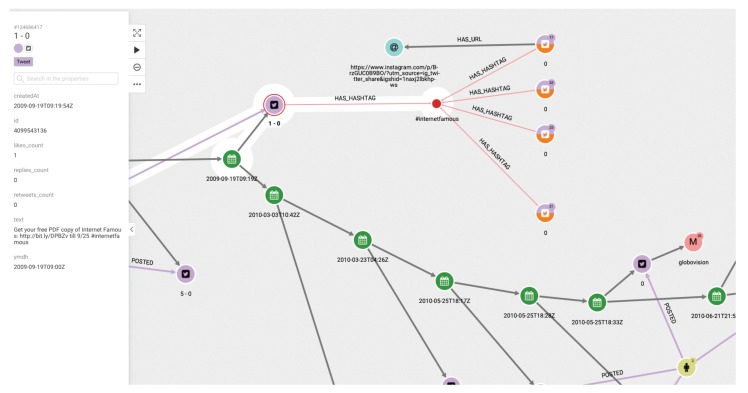
Tweets and hashtags in a timeline.

**Figure 6:**
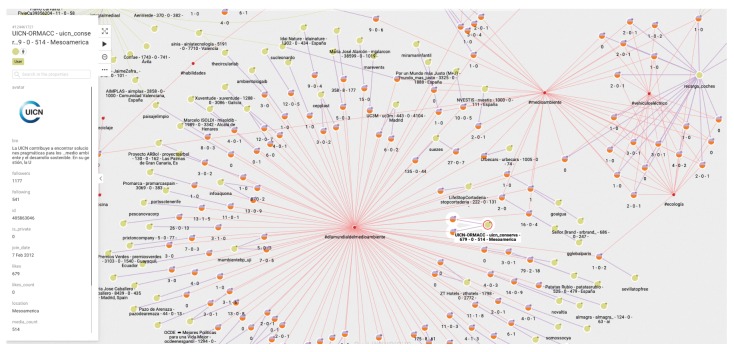
Global picture of a graph of tweets.

**Figure 7:**
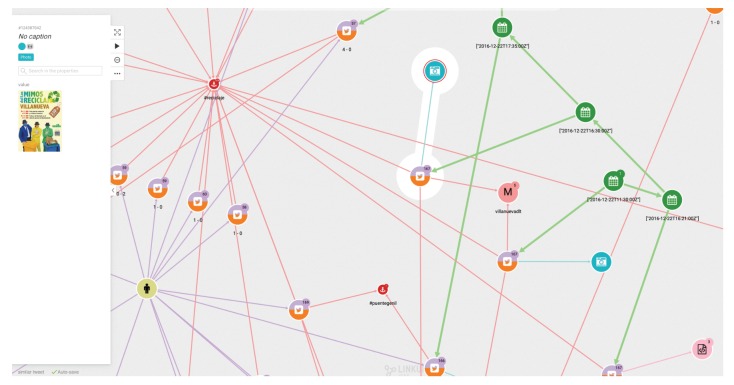
More elements for the analysis of the tweets network.

(1) First of all, we are interested in using the database generated as explained above to visualize the diffusion of the tweets of a given chain. This should be the starting point of an analyst’s study. Figure 3 shows the graph structure of the dissemination of tweets related to a specific topic. The green dots indicate the timeline. At each particular time, a tweet, represented by a pink dot, is generated. The yellow dots represent who posted the tweet, and the arrows between them indicate who follows whom. It is easy to see when the same user has posted more than one tweet, as more than one arrow leaves from the yellow dot representing that user.
(2) In a second step, the analyst could center the attention on the relation among concrete elements of the system. Figure 4 shows the relationships between two users. The system also allows us to show the graph of relationships between users (following, followers).
(3) The platform can show the tweets network for a given hashtag. The same relational graph can be enriched to show all the elements that could be considered relevant (Figure 5).
(4) A general representation of tweets related to a specific topic can be performed, as in Figure 6, which shows all tweets together with users and other elements related to the topic of “reciclaje”. The analyst can start looking at any of the vertices and find all the connections, but this is the general aspect of the graph when the researcher starts the analysis. When a vertex is clicked—for example, the one in the center—a new screen appears containing the local structure of the graph together with the labels of vertices and arrows as shown in Figure 7. Once the graph data-base is ready, the analyst can easily get general and partial pictures of the graph, and follow the links to study the sensitive parts. For example, he can start looking at any of the vertices and find all the connections, as in the example explained before. When a vertex is clicked—for example, the one in the center—a new screen appears containing the local structure of the graph together with the labels of vertices and arrows as shown in Figure 7.
(5) Further details regarding relationships and new elements can also be obtained. In Figure 7, the introduction of pictures into the tweet chain is also shown, together with the user who originated the action.
(6) The general schema of a given set of information can also be visualized (Figure 6). Although it is difficult to work with such representation, it illustrates the complexity of the starting point for analysis, and the analyst can then navigate the graph to perform their own study.

As we have seen, the final result of the application of the graph based database is a formal structure, that is sometimes called a knowledge graph. It must be understood that such a knowledge graph is defined as dynamic, in the sense that the relationship between vertices and edges is time-based. This is, in fact, one of its main properties, since it allows to adapt its structure at any time and update the information existing in the graph.

To finish this section, let us illustrate now the process of detection of fake news in a very concrete case. It shows one of the possible situations in which an analysis based on the graph network that have been explained can be performed. In this case, we use as main analytic element the visualization of the high rate of difussion of a given information, that is primary classsified as posibly fake. It would follow the following steps.

**Figure 8:**
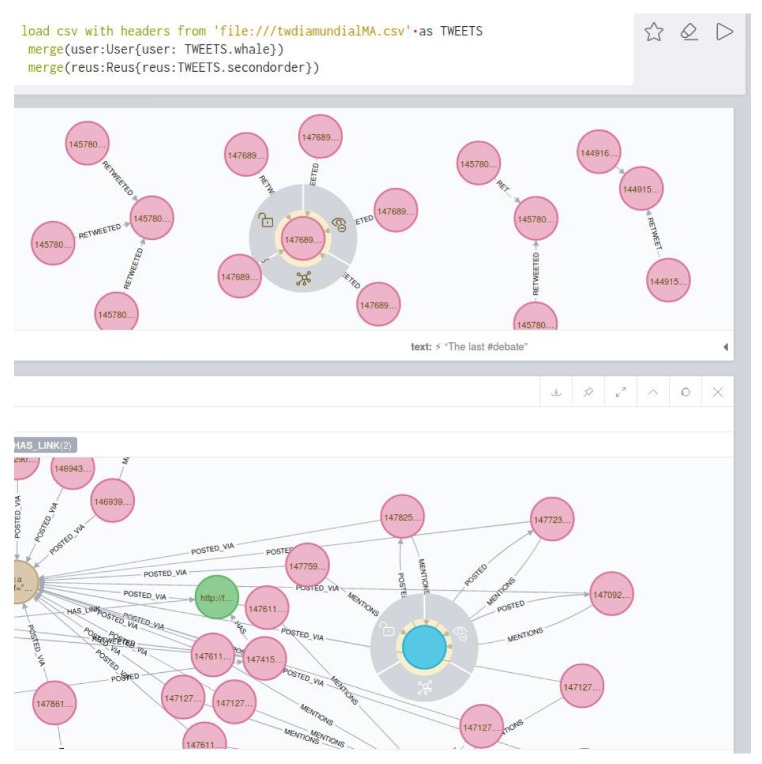
A step in the analysis of the tweets related to the dead whale problem.

(1) In the global representation of the graph in a given moment, a tweet with a lot of retweets of first-level and second-level is found. This can be seen just having a look to the general graph of the topic selected. The analyst centers its attention on it.

(2) The tweet reports the detection by the police of a dead whale in a western Mediterranean port. The tweet essentially contains the following information: “A dead whale has been found in the port of X. Due to the wounds on the animal, it seems that it has been killed by a boat. This coincides with the presence of a large ship from a major environmental organization off the coast.”

(3) Few of the users receiving the first-level retweets are detected by the analyst as “suspicious”, because of they have been already involved in other cases of difussion of fake news, or they are detected already as active elements of any global boot-based process.

(4) In the following step, the number of second-level retweets increase exponentially. Among the second-level users involved, some active ones that have been already detected in other cases as critics with the protection of the enviroment are found.

(5) The analyst formally classify the tweet as “potentially false”, and this finisehes the main graph based analysis. More sources of information are required at this stage. Also, tweets with the same label, but strongly questioning the validity of the information, start to appear. The users that send them have been already classified as fiable. In Figure 8, the reader can find a representation of the associated graph when the analyst focuses on the study of retweets.

As a final observation, let us note that there are some elements in the process described that show that some automatic analysis is possible. For example, if a set of users can be identified as bots, or as suspicious news sources, the structure of the graph can be adapted to automatically use this information.

## 4. Discussion

Our research on how to organize the information contained in a set of tweets leads us to propose the following analytical scheme, which is one of the main results of our study. The analysis of some specific tweet chains, together with some experiences on how to link them in a productive way, allows the creation of a systematic way of proceeding with a general analysis of false news and false information, which could also be applied to the public health context.

Firstly, it should be noted that the original selection of information items in the database is fundamental since it conditions the quality of the results obtained, and also because it delimits the general framework and limits of the study. This affects the first point of the procedure proposed below. This main rule also conditions the following steps of the analytical process. After this point, the analyst has to find by himself the main stream(s) of tweet chains, and restrict the attention to those with anomalous diffusion patterns. This is the part of the proposed method that could be automated —as we have explained in the Introduction— probably following different algorithms depending on the specific “pathological” properties being searched for.

Thus, as we have explained, the way we define the structure of the knowledge graph can also be explained as a dynamic process with the following sequence of steps:

- Consider a set of information items (for example, tweets), *A*, chosen according to certain selection criteria. The properties of the set, the relationships between its elements and the structure of the labels to be considered have to be chosen by the analyst in advance. After that, the original data set —for example, a csv file— can be loaded as explained in Section 2.3.
- The application of our method then follows with the transformation of *A* in a graph $G_{A}$, that is implemented in a graph database. The set of vertices $V_{0}$ is primary and produced by the users of the information source (Twitter, etc.) that send and receive the original set A. New vertices can also be added to the original set $V_{0},$ considering for example relevant tweets as vertices, hashtags or other elements that would become relevant relational items in the graph. The edges, E, and arcs, R, that connect them have their own properties, obtained from the relational information provided by the information source, for example, the direction of dissemination, or by intrinsic properties of the nodes. The system is ready to be used.
- The primary graph $G_{A}$ is then enriched by introducing new elements, following the criterium of the analyst. For instance, in the context of Twitter, followers of the users in $V_{0}$ or the users that follow the elements of $V_{0}$ can be introduced. Alternatively, if $V_{0}$ is taken in the time $t=t_{0},$ the set $V_{1}$ can be defined in the same way using the information in $t=t_{1},$ and a new graph can be defined by means of the information set $A_{1}=A_{t_{0}}\cup A_{t_{1}},$ where $A_{t_{0}}=A$ and $A_{t_{1}}=A_{1}.$ This would provide a rule for producing a dynamic graph.
- The graph structure implemented in this way in Neo4j is the play-ground for the analyst, and is the basis for a heuristic procedure. It can be used for studying some particular branches in the graph $G_{A},$ for example, by selecting the subgraph defined by the existence of certain keywords in the text of the information items or following the relational subgraph that begins in a particular dubious vertex. The resulting structure is not yet an algorithm for automatic detection of fake news, but a support tool for analysts.
- In a subsequent step, once the information has been structured in this way, certain algorithms can be implemented to provide lists of dubious sources of information, fake news, baseless rumours or gossip. Some of these algorithms —as tools followed by the analyst—have already been used for the study explained in Section 3; their implementation as software has not been done yet, and is the next step in our research project. As we have said, this would be carried out from at least two different points of view:
  (1) By defining algorithms based on dynamic properties of fake news dissemination (e.g., “local explosion” of an information item, detection of dubious original sources); or
  (2) By training a neural network or any other approximation procedure on the dissemination behaviour of false information with a big data set of dynamic graphs associated with fake news recognition.
  The diagram presented below shows the scheme of our technique. 
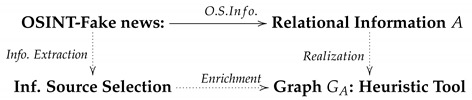
  As a result of our methodology, we are able to build an analytical structure in which particular searches can be performed; specifically, hashtags can be used to find sub-graphs in which the desired structured information is organized. Finally, the completed graph can also use semantic items to enrich its structure. For example, using random walks in the graph, we can define a proximity relation between concepts that allows us to characterize the semantic field of a linguistic expression. This can be understood as a method for providing a topological structure to the graph, on the basis of the proposed analytical tool (see References [12,15]).

## 5. Conclusions

We have presented a new method based on graph databases for the analysis of news dissemination on the Internet using open source information. In particular, we have focused our attention on the case of tweets generated about a rapidly spreading phenomenon that can occur on issues affecting public health. Our work is of a technical nature, and aims to show a general analytical approach to the study of the processes of dissemination of socially sensitive information. Our idea was to produce a graph structure with data extracted from tweets containing information on a given topic of current importance and the corresponding comments. The technique can be used for any information process with certain specific properties, such as rapid diffusion, unknown and dubious original sources, a large group of interested persons, or a high probability of fraud. This is the case, for example, with news about non-standard drugs in relation to healthcare, about current environmental issues, or on the so-called anti-vaccination movement. We present a specific example that shows the graph structure of the dissemination of tweets related to World Environment Day 2019 in order to show how the graph can be used to analyse the process from several perspectives, by following, for example, a specific hashtag or tweet chain.

The proposed platform could serve as the basis for an heuristic system for analysis of dynamic information such as (fake) news and other internet-based information frameworks. We believe it could also be the starting point for the implementation of automatic algorithms for the detection of fake news in healthcare. More advanced tools based on artificial intelligence—specifically, reinforcement learning-based distance metric techniques—would be needed. Some explanations are also provided about some open projects for the automation of search algorithms on the platform and the direction in which future developments could be carried out.
